# C-type natriuretic peptide prevents angiotensin II-induced cardiac remodelling and dysfunction

**DOI:** 10.1186/2050-6511-16-S1-A78

**Published:** 2015-09-02

**Authors:** Konstanze Roloff, Franziska Werner, Marco Abeßer, Katharina Völker, Hideo A Baba, Kai Schuh, Michaela Kuhn

**Affiliations:** 1 Institute of Physiology, University of Würzburg, Würzburg, Germany; 2 Institute of Pathology, University Hospital of Duisburg-Essen, Germany

## Background

Hypertensive cardiac remodelling is a major risk factor for cardiovascular morbidity and a leading cause of chronic heart failure. The activation of the renin-angiotensin system plays an important pathophysiological role in hypertensive cardiac remodelling [1].

C-type natriuretic peptide (CNP) belongs to the natriuretic peptide family. In the cardiovascular system, CNP is secreted from endothelial cells and possibly from cardiac fibroblasts, to act as autocrine/paracrine hormone [2]. It activates the guanylyl cyclase B (GC-B) receptor which synthesizes the second messenger cGMP. *In vitro* the CNP/GC-B pathway inhibits the proliferation and collagen synthesis of cardiac fibroblasts [3]. Therefore, we aimed to study the cardiac effects of synthetic CNP on Angiotensin II (Ang II)-induced cardiac fibrosis and hypertrophy *in vivo*.

## Methods and results

In primary cultured murine cardiomyocytes and cardiac fibroblasts, CNP induced strong and concentration-dependent increases in intracellular cGMP contents. In comparison, the cGMP responses to ANP were much smaller. To analyze CNP effects on Ang II-induced arterial hypertension and cardiac remodelling, 2-months old male C57/Bl6 mice were treated with vehicle (saline), CNP, Ang II or Ang II combined with CNP (12 mice per group). Osmotic minipumps filled with Ang II (delivery of 2000 ng/Kg BW/min) and/or CNP (50 ng/Kg/min) or vehicle were implanted subcutaneously during 2 weeks.

Infusion of Ang II provoked significant increases in diastolic (by 12 ± 2 mmHg) and systolic blood pressure levels (by 38 ± 3 mmHg; tail cuff measurements in awake mice). These hypertensive effects were accompanied by significant left ventricular (LV) hypertrophy (with enhanced LV weight/BW and enlarged myocyte diameters), LV interstitial fibrosis (quantified in sirius red stained LV sections) and enhanced mRNA expression of the hypertrophy marker brain natriuretic peptide (BNP, determined by qRT-PCR). Notably, CNP did not alter baseline blood pressure levels or the hypertensive reactions to Ang II. However, the peptide markedly and significantly prevented the cardiac hypertrophic and profibrotic actions of Ang II, as demonstrated at the organ, cellular and molecular (BNP) level.

To evaluate cardiac contractile functions, LV pressure-volume relationships were recorded by LV catheterization in anesthetized mice. LV contractile and relaxation functions of Ang II-treated mice were only mildly altered, as evidenced by subtle changes in LV diastolic pressures, contraction/relaxation rates, ejection fractions and stroke work. LV end-systolic pressures were enhanced, consistent with the enhanced afterload. Remarkably, CNP did not alter baseline LV hemodynamics but significantly improved LV contractility of Ang II-treated mice. Lastly, LV mRNA expression levels of the fibrosis markers collagen I and connective tissue growth factor (CTGF) were analyzed by quantitative real time RT-PCR. Ang II provoked ~2.5-fold increases in collagen I and CTGF. Simultaneous infusion of CNP significantly prevented the increases in collagen levels and had no effect on CTGF.

## Conclusion

In our experimental study infusion of a low dose CNP largely prevented the deleterious structural cardiac changes which follow neurohormonal activation by Ang II. These anti-remodelling effects of CNP possibly account for the improved cardiac contractile functions. Strikingly, these cardiac protective effects of CNP were fully blood pressure independent. The view that augmentation of cyclic GMP signaling in general benefits heart failure patients is supported by the recent clinical observation that a drug combining blockade of the Ang II/AT_1_-receptor with inhibition of neprilysin, a peptidase which degrades ANP and BNP, diminished the risks of hospitalization and death [3]. The here presented and other published experimental observations [4] suggest that the CNP–cGMP pathway may also represent a target for heart-protecting therapies.
